# Mechanisms of Iodide–Triiodide Exchange Reactions in Ionic Liquids: A Reactive Molecular-Dynamics Exploration

**DOI:** 10.3390/ijms20051123

**Published:** 2019-03-05

**Authors:** Aaron Byrne, Eduardo M. Bringa, Mario G. Del Pópolo, Jorge J. Kohanoff, Vanesa Galassi, Niall J. English

**Affiliations:** 1 School of Chemical and Bioprocess Engineering, University College Dublin, Belfield, Dublin 4, Ireland; aaronbyrne00@gmail.com; 2 CONICET & Facultad de Ciencias Exactas y Naturales, Universidad Nacional de Cuyo, Mendoza M5502, Argentina; mdelpopolo@gmail.com (M.G.D.P.); vanegalassi@gmail.com (V.G.); 3 CONICET and Facultad de Ingenieria, Universidad de Mendoza, Mendoza M5500, Argentina; 4 Atomistic Simulation Centre, School of Mathematics and Physics, Queen’s University Belfast, University Road, Belfast BT7 1NN, Northern Ireland; j.kohanoff@qub.ac.uk

**Keywords:** Bond exchange, reactive molecular dynamics, iodide, triiodide, charge transfer, energy barrier, diffusion

## Abstract

Efficient charge transport has been observed in iodine-doped, iodide-based room-temperature ionic liquids, yielding high ionic conductivity. To elucidate preferred mechanistic pathways for the iodide ($I^{-}$)-to-triiodide ($I_{3}^{-}$) exchange reactions, we have performed 10 ns reactive molecular-dynamics calculations in the liquid state for 1-butyl-3-methylimidazolium iodide ([BMIM][I]) at 450 to 750 K. Energy-barrier distributions for the iodine-swapping process were determined as a function of temperature, employing a charge-reassignment scheme drawn in part from electronic-structure calculations. Bond-exchange events were observed with rate-determining energy barriers ranging from ~0.19 to 0.23 ± 0.06 eV at 750 and 450 K, respectively, with an approximately Arrhenius temperature dependence for iodine self-diffusivity and reaction kinetics, although diffusion dominates/limits the bond-exchange events. This charge transfer is not dissimilar in energetics to those in solid-state superionic conductors.

## 1. Introduction

Room-temperature ionic liquids (RTILs) are molten salts with low volatility, comprising of molecular cations and anions having a melting point below 100 °C. They possess rheological, chemical and electrical properties making them flexible and viable alternatives to existing solvent-based systems. RTIL-based applications range from electrolytes for solar cells [1,2], fuel cells [3], batteries and supercapacitors [4], heat-transfer liquids and lubricating agents [5], and “green” solvents for catalysis and synthesis applications [6]. High ionic conductivity constitutes a particular desideratum, leveraged in dye-sensitised solar cells (DSSCs) so that a redox electrolyte (typically $I^{-}/I_{3}^{-}$) leads to ongoing charge renewal via electron-hole transfer from the dye to the electrolyte, whilst injected electrons travel through the semiconductor into the external circuit, until these charge carriers close the circuit by recombining at the cathode [7]. In particular, the extension of DSSC lifecycle to a near-indefinite extent by substituting a volatile electrolyte solution by a low-volatility solvent, usually an iodide-based RTIL, is a tantalising prospect [8,9,10].

RTILs’ outstanding charge-transport efficiency upon iodine addition to iodide-based systems [11,12,13,14,15,16,17], as compared with similarly viscous systems, appear predicated upon a bond-exchange mechanism [17], which is redolent of, in large part, the Grotthuss mechanism for protons’ diffusion in water [18]. This has been advanced as an explanation of molten polyiodides’ high electrical conductivity [19,20]. Wachter et al. have presented observations of non-Stokesian charge-transport behaviour in binary mixtures of 1-methyl-3-propylimidazolium iodide ([MPIM][I]) and 1-ethyl-3-methylimidazolium tetrafluoroborate ([EMIM][BF_4_]) [21]; moreover, Bentley et al. gauged charge transfer in 1-ethyl-3-methylimidazolium bis(trifluoromethanesulphonyl)imide ([C_2_MIM][NTf_2_]) as consistent with a Grotthuss-style bond-exchange processes [22].

Recently, Density Functional Theory (DFT) has proven an important tool in investigating $I^{-}/I_{3}^{-}$-charge-transfer mechanisms in RTILs, both for gas-phase cation-anion clusters [23] and in the extended solid state under periodic boundary conditions (PBC) [24], primarily from nudged-elastic-band-type calculations as a function of simple reaction coordinates. Thapa and Park determined limiting bond-exchange potential-energy barriers of ~0.8–1 eV for 1-ethyl-3methylimidazolium (EMI) clusters with iodine ions, depending on inter-ion separations [23]; Grossi et al. found a limiting barrier of ~0.5 eV in solid-state 1-butyl-3-methylimidazolium iodide ([BMIM][I]) with added iodine [24]. Although DFT acts as both a sensible and a computationally tractable initial foray into elucidating charge transport in disordered, liquid or glassy, iodide-based RTIL, fully deterministic (unbiased), long-time molecular dynamics (MD) of iodine-“doped” RTILs becomes a necessary, even essential, tool in the quest for sampling mass-transfer-limited bond-exchange reactions, where temperature-dependent diffusion limits intrinsic $I^{-}/I_{3}^{-}$-charge transfer, sampled over many RTIL configurations and phase space.

Here, using a forcefield applied in a variety of complex ionic systems [25], we have taken this further, in the sense of applying reactive forcefields and developing a charge-reassignment scheme drawn in part from electronic-structure calculations, we compute temperature-dependent charge-transfer energetics and kinetics in a [BMIM][I] liquid, with added iodine, wherein iodine anions couple and swap between $I^{-}$ and $I_{3}^{-}$ in the liquid state. This system was studied for $I^{-}/I_{3}^{-}$-charge transfer in the solid state by DFT by Grossi et al. [26], and we opt to examine this for ease of comparison with that work.

## 2. Results and Discussion

Molecular-dynamics (MD) simulations were performed with this ReaxFF/charge-setting scheme for 10 ns at 450, 550, 650 and 750 K in the NVT ensemble with Nosé-Hoover thermostat (τ = 0.25 ps) on the respective MM-based MD configurations with the corresponding time-averaged densities.

**Figure 1:**
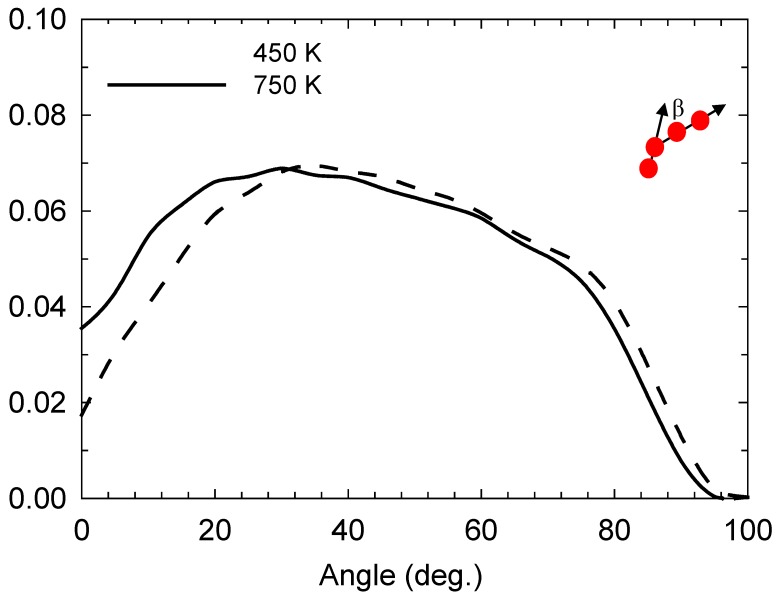
Normalised approach-angle (β) [24] distribution at 450 and 750 K; β (depicted schematically) is the angle with respect to the I_3_^−^I^−^ collinear axis [24].

**Figure 2:**
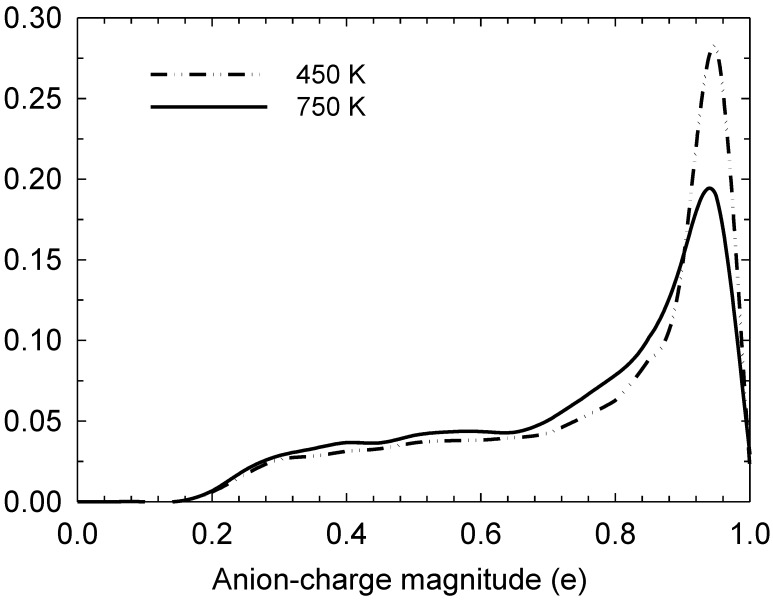
Normalised iodine-atom charge distribution at 450 and 750 K, computed for all iodine atoms in the simulation box.

In Figure 1, we observe that the reaction is angle-selective for the angle of approach with respect to the I_3—_-I^−^ collinear axis, with a higher incidence at angles between 20 and 40°. However, it occurs even at higher angles up to 80°. This selectivity is still preserved at high temperatures, such as 750 K, albeit with an increase in the incidence at angles below 20°. Figure 2 depicts the iodine-anion charge distribution at 450 and 750 K, which gives an approximate impression of the distribution between the relative populations of anion types. It can be seen that at higher temperatures, there is a greater propensity for anions to be in alternative states to I^−^, which is consistent with a higher number of bond-exchange events, as shall be discussed later.

Now, polyiodide species (e.g., I_5_^−^ and I_7_^−^) are known to be present in iodide-based RTIL at high I_2_ concentration. In any event, no heavier polyiodide species were observed on any routine basis, despite some recent AIMD simulations in ionic crystals suggesting occasional formation thereof [27]. However, this is hardly surprising, in that our investigation considers the case of a single I_2_ dopant molecule in the simulation cell, i.e., a very low concentration of I_2_, which prevents the formation of longer polyiodides for stoichiometric reasons.

**Figure 3:**
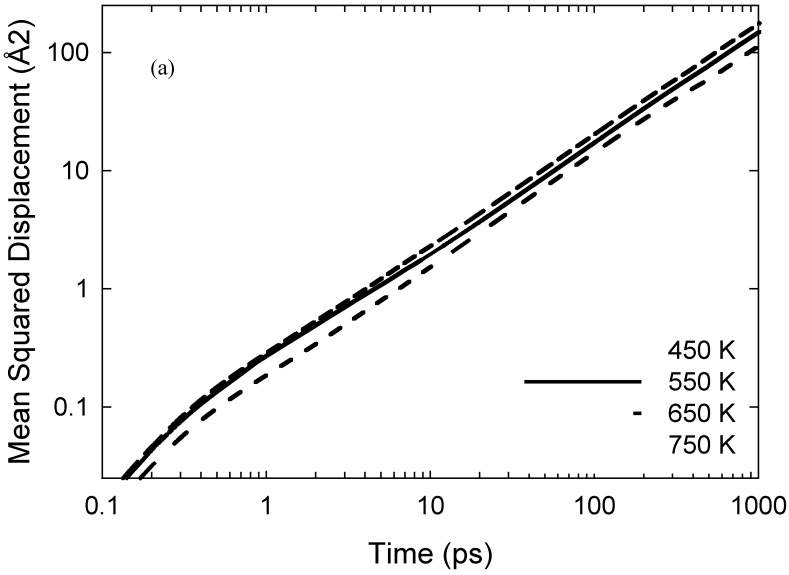

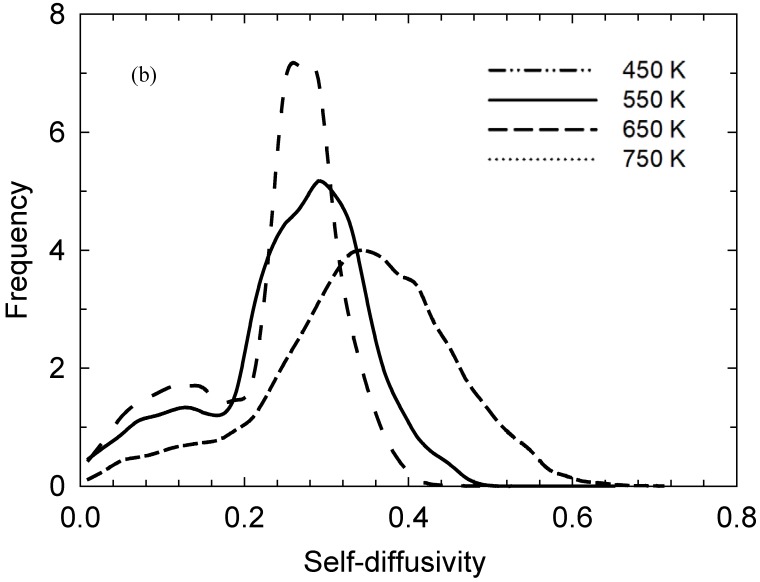
(**a**): Mean squared displacement (MSD) of the iodine atoms sampled over 10 ns, with log-log plot showing long-time/limiting slopes of ~0.9 and above, indicating the establishment of near-Fickian diffusion; Fickian diffusion results in a limiting log—log slope of unity. (**b**) Normalised probability distributions of individual iodine atoms’ self-diffusivities sampled from individual-atom MSDs’ limiting slopes over 0.25 ns periods. Here, depending on temperature, the sampling time is ~20–150 times the reaction events’ timescales, allowing for instances of lower diffusivities to be seen rather dramatically at 450 and 550 K with the “hump”, or shoulder, in the 0–0.2 × 10^−9^ m^2^/s region clearly evident when motion is slowed due to participation in bond-exchange reactions.

Figure 3a depicts the mean squared displacement (MSD) of the iodine atoms at each temperature (sampled over the full 10 ns trajectories); the log-log plots have long-time/limiting slopes of ~0.9, indicating establishment of essentially Fickian diffusion. Normalised probability distributions of individual iodine atoms’ self-diffusivities are shown in Figure 3b, sampled from individual-atom MSDs’ limiting slopes over 0.25 ns periods [28]. It is important to note that here, this MSD-sampling time was selected to be ~20–150 times the bond-exchange events’ timescales, although sufficiently long for adequate statistical sampling of such MSDs; this affords recording of rather dramatic instances of lower diffusivities at 450 and 550 K with the shoulder, or “hump”, in the 0–0.2 × 10^−9^ m^2^/s region clearly discernible when motion is slowed due to involvement in reaction events. Also, the distributions in Figure 3b are clearly non-Gaussian, with a tail at higher self-diffusivities and temperatures similar to that seen in [29] for individual water molecules in hydration layers.

**Figure 4:**
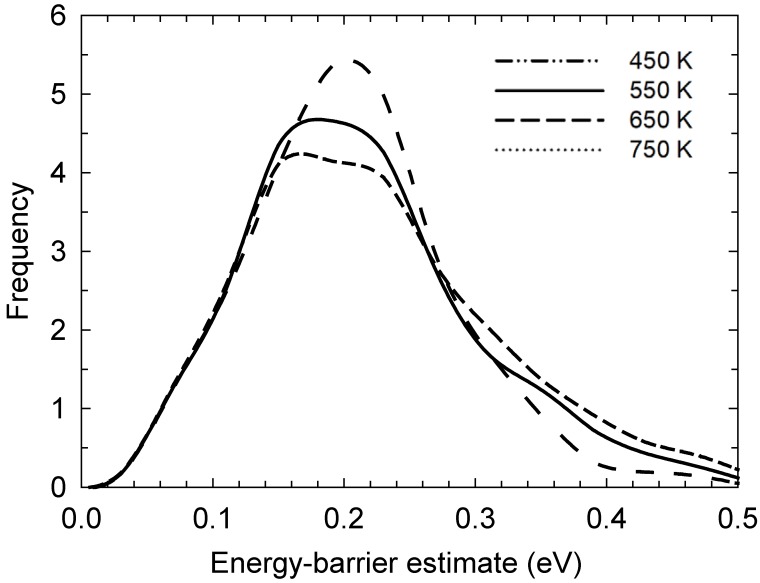
Normalised probability distributions of each reaction’s estimated potential-energy barrier, as sampled from consideration of participating iodine atoms’ interaction-energy variation immediately before and at the highest interaction-energy point during bond-exchange events. The move towards lower potential-energy barriers with increasing temperature is evident.

Normalised probability distributions of each reaction’s estimated potential-energy barrier are presented in Figure 4 at each temperature. This was gauged by considering each participating iodine atoms’ change in interaction energy (with each other and, more widely, the rest of the system) immediately before and at the highest interaction-energy point during bond-exchange events. However, to denote this as a “potential-energy barrier” is somewhat misleading: in truth, this neglects solvent-reorganisation energy, so this label is somewhat tentative. Still, for a bond-exchange reaction, most of the energy barrier originates from partially (e.g., half) -broken/-formed bonds, so this component of the interaction energy with each other tends to dominate over interactions with the wider system. Moreover, the high viscosity of these liquids hinders solvent reorganisation during the bond-exchange reaction, leading our model to be a somewhat reasonable approximation. In any event, bearing these qualifications on the concept of energy barrier, the move towards lower energy barriers with increasing temperature is evident, with values of ~0.19 to 0.23 ± 0.06 eV at 750 and 450 K, respectively, which is not unexpected. Indeed, this is rather consistent with other estimates of energy barriers in the liquid state, in the general ~0.2–0.3 eV region [12,13,14,15,16,17]. In a sense, the interaction-energy-variation metric is a ‘proxy’ measure for a free-energy barrier, in that it excludes potential-energy changes in the broader liquid as a result of bond-exchange reactions, in addition to ignoring entropy. However, the changes in the energy of the solvent itself as a result of these reactions is likely to be minor. We return to the more subtle matter of entropy anon.

In terms of the BMIM cations’ behaviour, it was observed, in essence, that they were largely an ‘innocent’ bystander in the bond-exchange reactions, and did not influence the such events to any great extent. There was no chemical reactivity recorded for these cations, and they behaved in a similar manner to the MM-based MD simulations carried out before the adoption of ReaxFF.

**Figure 5:**
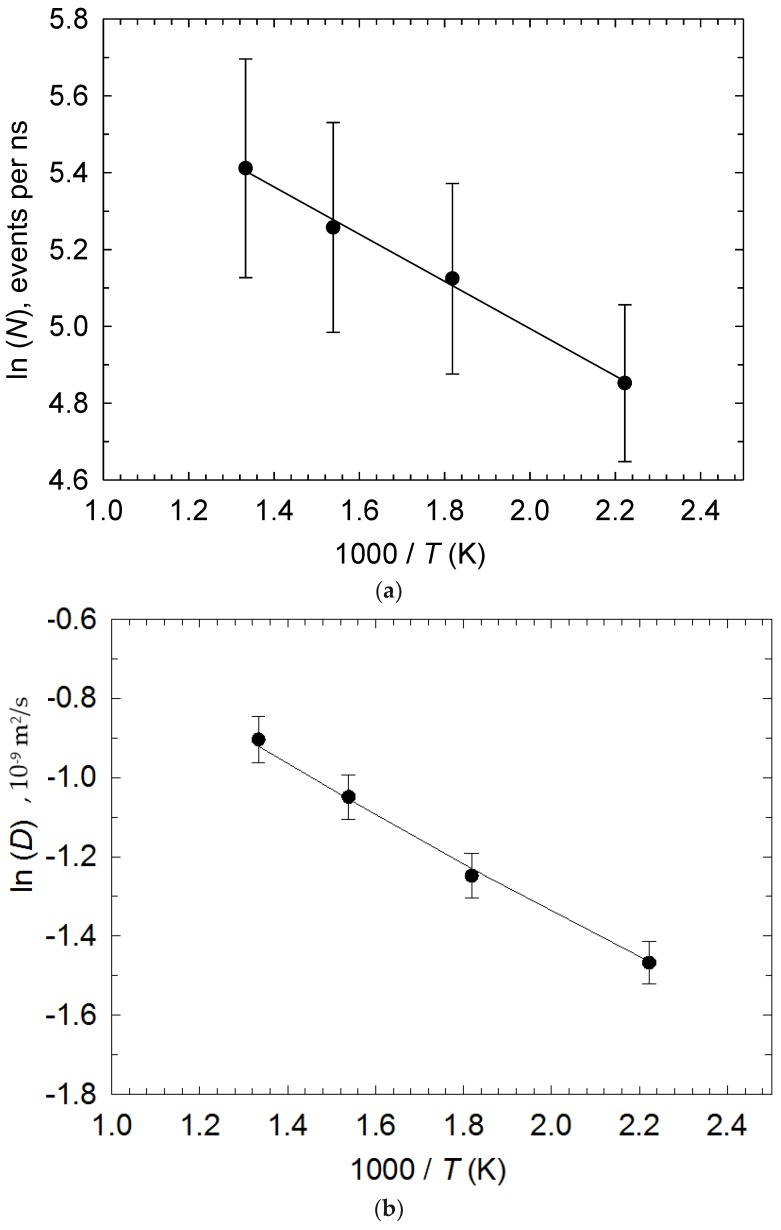
Arrhenius plots of (**a**) number of reactions per nanosecond, *N*, and (**b**) self-diffusivity, *D* (from Figure 3a), with error bars. The *r*^2^ of both linear-regression lines is over 99%. A Student’s *t*-test on the resulting *N*- and *D*- activation energies establishes non-rejection of *H*_0_ (see main text).

In Figure 5, Arrhenius plots are provided for both the number of reactions per nanosecond, *N*, and self-diffusivity [29], *D* (from Figure 3), with good fit. A Student’s *t*-test on the resulting *N*- and *D*- activation energies of 5.12 and 4.88 kJ/mol (or 0.053 and 0.048 eV), respectively, establishes non-rejection of the null hypothesis *H*_0_ with respective linear-regression activation-energy uncertainties of 0.43 and 0.41 kJ/mol. This shows, unambiguously, that the reaction kinetics are diffusion-limited (with a pre-exponential factor for *N* of 505 ns^−1^), or mass-transfer-controlled. Now, it must be understood that *D* is averaged over all anions, so its activation barrier contains two contributions–related to: (i) the “I^−^ caging” barrier experienced by the more abundant I^−^ ions when leaving their solvation cages in terms of Fickian diffusion as a thermally activated event, and (ii) the (less numerically abundant) bond-exchange process. Certainly, the vast majority of time is spent on diffusion as opposed to bond-exchange events.

**Figure 6:**
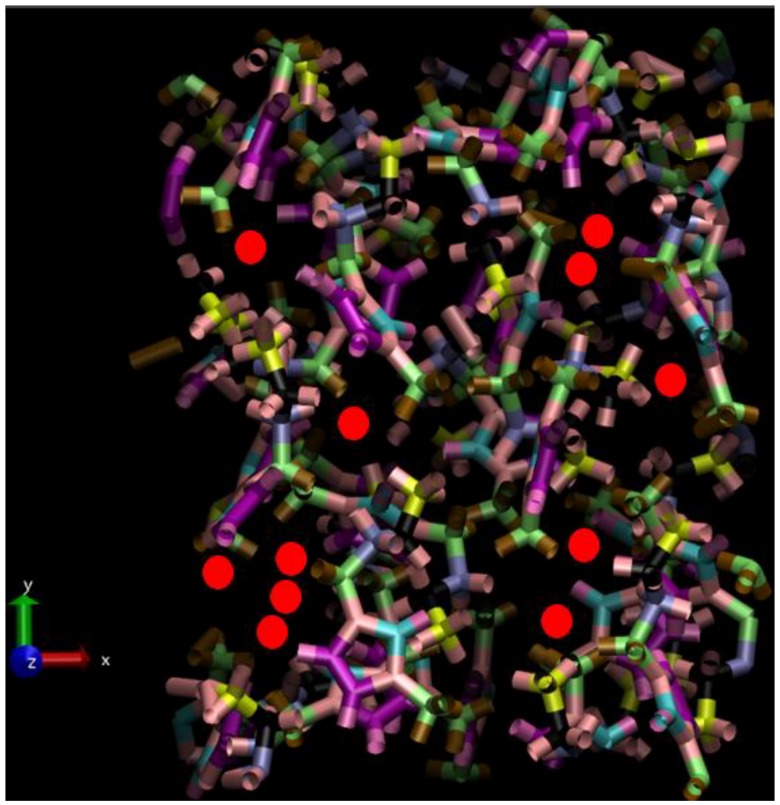
Representation of a typical liquid-phase configuration, alongside the laboratory Cartesian axes (with the z-axis perpendicular to the page); selected iodine anions are depicted as red spheres with their approximate ionic radius, and the cations are depicted in terms of their covalent-bonding motifs, in a “packed” manner across PBC simulation-box boundaries. Some of the shown iodine ions in the upper z-axis part of the simulation box are ‘poised’ in a pre-reactive configuration, ready for subsequent bond-exchange interaction.

In an effort to gain insight into the underlying reaction dynamics, without background diffusion limitations, we measured the average times taken for reactions to occur once poised in the initial state and immediately afterwards (taken as the time for charge to reach −0.67*e* from essentially −1*e* as the $I^{-}$ ion reduces its negative charge upon incorporation into the $I_{3}^{-}$ moiety). We obtained 9.78, 5.49, 2.94 and 1.71 ps at 450, 500, 650 and 750 K, respectively. Now, to be clear, ref. [17] estimates nanosecond bond-exchange events, inferred from experimental measurements. The obvious disparity here with respect to the simulation findings very likely stems from the fact that the present ReaxFF approach does tend to underpredict–systematically, so–energies compared to DFT by of the order of a third. If one assumes, arguably not unreasonably, that DFT is a more reliable approach, then lower energy barriers (Figure 6) would also be expected to result in faster reaction kinetics.

In any event, notwithstanding these just-mentioned timescale disparities and caveats, taking the inverse of these reaction times as a reaction rate and performing an Arrhenius fit, we obtain an activation energy of 16.2 ± 1.9 kJ/mol (or 0.168 ± 0.02 eV). Given the central tenets of reaction-rate theory [30], this is akin to a free energy for intrinsic bond-exchange reactions, which is close to the ~0.19 to 0.23 ± 0.06 eV range seen by the imperfect potential-energy-barrier ‘proxy’ measure, meaning that the entropy term may perhaps account for the non-negligible level of ~0.02–0.06 eV.

In terms of further consistency with experimental trends, it is instructive to compare iodine ionic self-diffusivities, at least in a high-level sense, with experimental apparent/charge-diffusivity observations of [13]. Specifically, the apparent-diffusivity results of [13] suggest that I_3_^−^ as an entity should have a somewhat larger diffusivity than I^−^. Preliminary analysis of the individual-atom MSDs indicates that the lower diffusivities in Figure 3’s probability distribution is typically dominated by iodides, particularly for the lower-temperature ‘humps’; this is consistent with [13]. Returning to the double contribution to *D*’s thermal-activation barrier (albeit with different abundances), as mentioned above, this somewhat lower intrinsic diffusivity typical of I^−^ indicates that a lower barrier would be expected in the absence of doping to the ionic liquid.

We used the Green–Kubo approach to compute the electric conductivity as ~9 ± 3 S/m at 450 K, which is the closest temperature to experiments [13,16,17]. This is perhaps ~5–10 times higher than the typical experimental range, but, then again, with faster kinetics than experiment [13,16,17] and lower energy barriers than DFT.

The iodide-triiodide exchange mechanism in the liquid phase is very similar to that observed in the solid phase of (BMIM)(I), in the sense that the entering and leaving iodides move in a concerted way and acquire similar partial charges as the reaction complex moves along the reaction coordinate. However, in the solid phase, the rigidity of the crystal lattice imposes certain restrictions on the angle of approach/departure of the entering/leaving I^−^, which forces the reacting I_3_^−^ to rotate before the bond-exchange process. The reorientation of I_3_^−^ in the solid phase imposes an energy penalty, as discussed in detail in [24], which is absent in the isotropic liquid phase.

## 3. Methods

As in ref. [24], a system of 32 [BMIM][I]– ion pairs was established as a 2 × 2 × 2 crystal-state supercell; adding an extra I_2_ molecule adjacent to an iodide ($I^{-}$) anion, forming a triiodide ion ($I_{3}^{-}$), led to an electroneutral orthorhombic simulation box comprising 834 atoms. Under PBC, respective *x*-, *y*-, *z*- box dimensions were 16.561, 21.577 and 23.997 Å (coinciding with *a*-, *b*-, *c*- crystallographic directions), consistent with ambient pressure [26]. To generate the liquid state via high-temperature MD melting, the Canongia-Lopes et al. molecular-mechanics (MM) potential was adopted for cations, ref. [31] whilst iodide ions were assigned a charge of −1e, and Lennard–Jones (LJ) 12–6 parameters of 0.07 kcal/mol and 5.4 Å. The charge on the (nominal) triiodide ion was equally distributed amongst the three atoms, thereby possessing $-\frac{1}{3}e$ each, with identical LJ treatment to iodide ions. The Ewald approach was applied for long-ranged Coulombic interactions [32]; the cut-off for all real-space interactions was 8 Å. MD was performed at 800 K for 5 ns in the NVT ensemble with a Nosé-Hoover thermostat [32] (τ = 0.25 ps); full melting to the liquid state was accomplished within less than ~1 ns. NPT-ensemble MD was performed subsequently for 5 ns at 450, 550, 650 and 750 K at 1 bar target pressure (featuring isotropic Anderson-Hoover cell variation, with τ*_P_* = 2.5 ps & τ*_T_* = 0.25 ps) [32]; the ensuing relaxed densities were some 5–11 % lower in the respective 450 to 750 K range (i.e., ~1.37, 1.35, 1.32 and 1.30 g/cm^3^, respectively) than the ambient-temperature experimental liquid-state value [33], but this is expected, naturally, due to thermal expansion. A typical liquid-phase configuration is shown in Figure 6.

**Figure 7:**
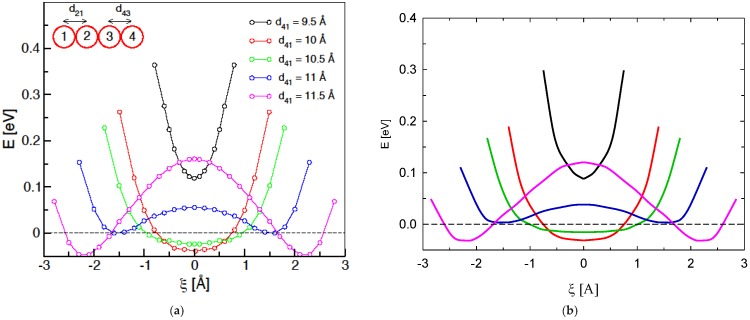
Gas-phase study of the system potential energy as a function of ξ, for various d_41_ separations, via (**a**) DFT single-point calculations (from ref. [24]) and, (**b**) the present ReaxFF/charge-reassignment scheme on the right; there is a legend and schematic on the (**a**) panel. A collinear arrangement is adopted for ‘head-on’ approach of 1 and departure of 4. For d_41_ is less than, or up to, ~10.5 Å, a single-energy minimum is evident for ξ = 0 (i.e., d_43_ = d_21_), associated with the formation of a symmetric $I_{4}^{2-}$. Once d_41_ exceeds ~10.5 Å, two minima develop, for I^−^····I_3_^−^ and I_3_^−^····I^−^ (the approach of I1 and departure of I4, respectively). Note: the (**a**) graph from ref. [24] is reproduced with the kind permission of the American Chemical Society.

Following these reassuring MM-based MD findings, we applied a reactive force-field approach (ReaxFF) [34] for MD with 0.5 fs step towards the relaxed liquid-state systems for all atoms (except in terms of partial charges vide infra); we judge ReaxFF as an adroit compromise between (putative) DFT accuracy and the speed of MM-(pairwise)-forcefield simulations, with the latter offering reasonable system-density results. In ReaxFF-based MD, all but non-bonding terms depend on the bond-order parameter, determined dynamically on the basis of inter-atomic distances, thus handling chemical changes [34]. Particular parameters, e.g., Lennard–Jones interactions, were taken from ref. [35], deemed suitable for general-purpose hydrocarbon systems. Although atomic charges are also typically reassigned dynamically in ReaxFF via charge-equilibration methods [36,37,38,39,40,41,42], these approaches break down rapidly for explicitly anionic-cationic pairs, e.g., in ionic-liquid systems, reducing the charge of each ionic moiety, in practice, to essentially nought. Within established, efficient ReaxFF implementations, for instance LAMMPS [43,44], there is currently limited scope and outlook for remedying this existential drawback [45]. Therefore, we retained cation partial charges of ref. [26], and, for iodines, we implemented the following charge-reassignment scheme during ReaxFF-based MD [33], via an interactive Python interface to LAMMPS at each MD step. The charge on each iodine atom, *Q*, is calculated as an empirical fit by calculating the distance to every other iodine atom (within an 8 Å cutoff), and finding the nearest two, with distances *r*_1_ (nearest) and *r*_2_ (next-nearest):

*Q* = *c*_1_ (*r*_1_/*r*_2_) (*r*_2_ − *r*_1_) + *c*_2_ (*r*_2_ − *r*_1_) *^c^*3 + *c*_4_*r*_1_*^c^*5 (1)

where multiplicative coefficients are *c*_1_ = −0.118, *c*_2_ = 0.105 and *c*_4_ = −0.167 and the exponents are *c*_3_ = 1.575 and *c*_5_ = 1.165; these coefficients were drawn form least-squares fittings of gas-phase calculations. A rebalance function then ensures overall charge neutrality by dispersing any excess negative charge throughout the remaining I^−^ anions of the system in a uniform way (i.e., regardless of position). Although heuristic, this scheme captures essential details in a reassuring manner: for reorientations [24], the central atom in a triiodide ion retains a charge of around $-\frac{1}{3}e$, and reaches ~$-\frac{1}{3}e$ upon alternating chemical “personality” during a bond-exchange event from the outer ion in contact with the approaching $I^{-}$ ion (with charge of ~$-\frac{1}{2}e$) [24]. Should the highly improbable event arise of the next-nearest iodine atom is outside of the 8 Å cut-off, *r*_2_ in Equation 1 is set at 8 Å. In practice, though, this event was never seen to occur during any of the simulations in the present manuscript. This putative “boundary condition” corresponds to having just one or two iodine atoms in a local region of anomalously low (liquid-phase) iodine density, i.e., essentially the gas phase situation; see Figure 6 for validation details with respect to handling the gas phase. Indeed, for validation of this ReaxFF/charge-reassignment scheme, there is semi-quantitative accord with (largely symmetric) Hirshfeld-charge evolution of participating iodine atoms on the DFT-simulated crystal-state bond-exchange events (cf. Figure 2 of [24]), using the provided configurations [24]; the final charge is ~−0.7 to −0.65*e* from essentially −1*e* as the $I^{-}$ ion reduces its negative charge upon incorporation into the $I_{3}^{-}$ moiety, which agrees with around −0.6*e* of ref. [24]. In ref. [24], PBE exchange-correlation was used in conjunction with Grimme’s D2 dispersion correction; all electrons (core and valence) were treated explicitly, with orbitals in a 6–31 G * basis set and electron density expanded in plane waves with a 350 Ry cut-off. This level of treatment was found to be satisfactory with respect to crystal-state density estimation [24]. Naturally, in the liquid state of the present study, the charge level upon transformation to the $I_{3}^{-}$ ion with the present scheme adopts a wider range of values, spread between ~−0.8 and −0.5*e*, with temperature dependence (see below). In terms of energetics, the scheme reproduces semi-quantitatively those of the “head-on” $I^{-}\cdots I_{3}^{-}$ bond exchange in vacuo (Figure 7); these are generally roughly ~70 % of the DFT-treatment energies. This may be because of the general-hydrocarbon nature of the ReaxFF parameterisation of [34] employed in the present work. In terms of the crystal state itself, the scheme’s predictions of potential-energy barriers for Figures 3 and 7 of ref. [24] (using that work’s configurations) are roughly two thirds of DFT levels, i.e., a limiting barrier of ~0.34 eV for non-collinear bond exchange [24].

In any event, having outlined the nature of the charge-reassignment scheme, we note that classical-MD simulations of ionic liquids are complex, and, in general, they involve large variation in fixing partial charges depending on the chosen parametrisation [46,47]. Indeed, recent force-fields involving charge exchange have not been yet parameterised for ILs [45]. Even ab-initio MD (AIMD) simulations of ILs are challenging, with deviations larger than 5 kJ/mol in the interaction energy along the entire potential-energy curve as obtained by various functionals in comparison to an accurate reference [48]. Because of all of these difficulties, and taking this as a first attempt at this difficult charge-transfer/definition problem with a heuristic, less formal approach, additional forces due to this charge exchange of Equation. 1 were not included. Rigorous energy conservation, when simulated in the NVE ensemble, does not therefore happen; here, the total system energy experiences fluctuations of the order of 0.5 kJ/mol, which is about double the level of energy fluctuations due to the Verlet integrator for the time step used. However, all of our ‘production’ simulations have been performed under NVT or NPT conditions. We feel that this is an acceptable level of approximation in practice to initially tackle this inherently difficult problem, given IL force-fields’ shortcomings; ref. [45,46,47,48] no doubt, more refined approaches can, should and will be adopted by the reactive-event simulation community in the future. This more promising outlook and prospect is explored to some extent in the Conclusions section.

## 4. Conclusions

A consistent picture of charge transfer in the liquid emerges, unlike the Grotthuss-type mechanism in the crystal state found in ref. [24]. Indeed, using Grotthuss-decomposition analysis along the lines of ref. [49] for the MSDs, it appears that self-diffusivity is dominated (~85%) by non-Grotthuss contributions. However, the energy barriers agree reasonably well with previously estimated rate-limiting energy barriers of ~0.2 to 0.3 eV [12,13,14,15,16,17]. In fact, mechanistically, diffusion dominates/limits the bond-exchange events, so the physical liquid-state picture is akin to a classical diffusion-limited elementary chemical reaction. Clearly, ReaxFF with well-designed charge reassignment can act as an efficient prototype method for simulation of various RTILs of potential industrial interest, as a cost-effective ‘screening tool’, such as candidate electrolytes for DSSCs [8] In view of the large energy barriers determined here, and the lack of perfect fit of the heuristic charge-reassignment fit with Hirshfeld charges [24], biased ab-initio MD methods employing QM/MM approaches may be another promising avenue to offer further success. However, as we have discussed, charge-reassignment schemes need to be calibrated carefully with respect to their performance [36,37,38,39,40,41,42]; as mentioned previously, handling explicit ions using ReaxFF charge equilibration is very challenging, motivating us to develop our own heauristic scheme in the present work. Certainly, the advanced eReaxFF scheme of ref. [42], with its integration of atom-condensed Kohn-Sham DFT charge calculations into ReaxFF to mimic some salient and important features of Ehrenfest dynamics, signals that new and promising directions are becoming available for handling the liquid- and solid-phase reactivity if ionic species, especially for larger-scale systems. However, other exciting methods, such as interactive machine-learning and neural-network techniques to compute optimal ionic charges “on the fly” in MD based on ReaxFF or QM/MM would also be worth exploring in the future.
